# Clinico-Radiological Profile, Management Strategies, and Survival Outcomes in Medulloblastoma: A Retrospective Study From a Tertiary Care Center

**DOI:** 10.7759/cureus.106056

**Published:** 2026-03-29

**Authors:** Jason Golmei, Bal Krishna Ojha, Chhitij Srivastava, Ankur Bajaj, Awdhesh Yadav, Anit Parihar, Shivanjali Raghuvanshi, Kirti Srivastava, Shweta Dubey

**Affiliations:** 1 Neurosurgery, King George’s Medical University, Lucknow, IND; 2 Interventional Radiology, King George’s Medical University, Lucknow, IND; 3 Pathology, King George’s Medical University, Lucknow, IND; 4 Radiotherapy, King George’s Medical University, Lucknow, IND; 5 Radiodiagnosis, King George’s Medical University, Lucknow, IND

**Keywords:** adjuvant therapy completion, low- and middle-income countries, medulloblastoma, prognostic factors, survival

## Abstract

Background

Medulloblastoma is the most common malignant pediatric brain tumor. While survival outcomes have improved in high-income countries, results in low- and middle-income countries (LMICs) remain limited by delayed presentation, socioeconomic constraints, and incomplete adjuvant therapy. Hence, this study aimed to evaluate the clinico-radiological profile, management strategies, and prognostic factors influencing survival in patients with medulloblastoma treated at a tertiary neurosurgical center.

Methodology

A total of 56 patients with histologically confirmed medulloblastoma operated on between January 2019 and March 2024 were retrospectively analyzed. Clinical, radiological, surgical, and treatment data were collected. Survival was estimated using Kaplan-Meier analysis and compared using the log-rank test. Multivariate Cox proportional hazards modeling was performed using a parsimonious model.

Results

The mean age was 9.7 ± 4.3 years (range = 3-28), with 40 (71%) males. Most tumors were vermian in location (50, 89.3%) and ≥50 mm in size (38, 67.9%). Gross total resection was achieved in 47 (83.9%) patients. Completion of adjuvant therapy was associated with improved survival (median 23 vs. 4 months; p < 0.0001).

Conclusions

In this LMIC cohort, completion of postoperative adjuvant therapy (craniospinal radiotherapy followed by chemotherapy) emerged as an important modifiable factor influencing survival. The association between ataxia and improved survival reflects earlier detection rather than biological advantage. Strengthening postoperative radiotherapy compliance and multidisciplinary coordination is essential to improving outcomes.

## Introduction

Medulloblastoma is the most common malignant brain tumor of childhood, accounting for nearly one-fifth of all pediatric central nervous system neoplasms and 40% of posterior fossa tumors [1,2]. Though primarily a pediatric disease, around 20% of cases occur in adolescents and adults, in whom biological behavior and therapeutic response differ substantially [3,4]. The integration of advanced imaging, microsurgical resection followed by craniospinal irradiation, and risk-adapted multi-agent chemotherapy has improved five-year overall survival to 70%-85% in high-income countries (HICs) [5,6].

In low- and middle-income countries (LMICs), survival remains below 50%, reflecting late diagnosis, socioeconomic barriers, and incomplete access to adjuvant therapy [7-9]. Delays in referral and limited access to specialized care contribute to larger tumor size and advanced presentation at diagnosis [10,11]. Indian series have highlighted that even when gross total resection (GTR) rates match global standards, completion of adjuvant therapy, particularly radiotherapy, remains a major contributor to survival [12].

This study analyzes the clinico-radiological profile, management strategies, and survival outcomes of 56 patients with medulloblastoma treated at a tertiary neurosurgical center. It focuses on the relative prognostic contributions of tumor size, extent of resection, histopathology, and adjuvant therapy completion in an LMIC setting.

## Materials and methods

This retrospective observational study included 56 consecutive patients with histologically confirmed medulloblastoma operated on between January 2019 and March 2024 at a tertiary neurosurgical center. Inclusion criteria were definitive tumor resection for medulloblastoma with available clinical, imaging, treatment, and survival data. Exclusion criteria included biopsy-only or palliative procedures, because survival analysis required definitive tumor resection followed by adjuvant therapy, as well as incomplete clinical records. Patients were clinically stratified into standard-risk and high-risk groups based on age, extent of resection, and presence of metastasis, where data were available. The study received Institutional Ethics Committee approval (Ref: XXI-PGTSC-IIA/P70).

Preoperative MRI was performed in all patients and used to characterize tumor origin (vermis, hemisphere, or cerebellopontine angle), maximal diameter (dichotomized at 50 mm), and enhancement pattern (homogeneous vs. heterogeneous). Hydrocephalus was present in the majority of patients at presentation. Cerebrospinal fluid (CSF) diversion was performed in 42 (75.0%) patients before definitive tumor surgery, predominantly in the form of ventriculoperitoneal shunt placement. Many patients presented with features of raised intracranial pressure requiring urgent intervention. In such cases, CSF diversion was performed before complete imaging workup, followed by definitive tumor resection once clinically stabilized and appropriate imaging was completed. Early postoperative MRI was not routinely feasible due to logistical constraints; therefore, CT-based criteria were used for standardized assessment of the extent of resection. A postoperative CT scan within 72 hours was performed in all patients and used to determine the extent of resection using objective area-based thresholds. GTR was defined as no visible residual tumor. Near-total resection (NTR) was defined as residual tumor ≤1.5 cm² in the maximal cross-sectional area. Subtotal resection (STR) was defined as residual tumor >1.5 cm² or a clearly measurable mass remnant. These thresholds were adopted to standardize assessment where early postoperative MRI was not feasible. Early CT-based assessment of the extent of resection has also been described in resource-limited settings where immediate postoperative MRI is unavailable.

Postoperative complications were prospectively documented using uniform definitions and managed per protocol. Meningitis required clinical suspicion plus CSF confirmation and was treated with targeted antibiotics with CSF diversion, as indicated. Postoperative hemorrhage was diagnosed on CT and managed conservatively or surgically based on mass effect. Lower cranial nerve palsy was defined by persistent dysphagia/hoarseness requiring airway and nutrition support. Cerebellar mutism was diagnosed in children with postoperative speech arrest and treated with steroids and rehabilitation.

Adjuvant therapy consisted of craniospinal irradiation delivered to a dose of 36 Gy in 20 fractions, followed by a posterior fossa boost of 18 Gy in 10 fractions, achieving a total dose of 54 Gy. This radiotherapy protocol was applied uniformly across patients in this cohort as per institutional practice. Chemotherapy consisted of platinum-based multi-agent regimens (commonly including cisplatin, vincristine, and cyclophosphamide), administered as per institutional protocol. While the exact number of cycles varied depending on tolerance and follow-up, chemotherapy was generally planned following completion of radiotherapy. Chemotherapy was planned for all eligible patients as part of standard combined modality treatment in accordance with institutional protocol.

Adjuvant therapy completion was defined as completion of planned craniospinal irradiation (≥30 fractions) followed by adjuvant chemotherapy according to institutional protocol. Non-completion included early discontinuation or failure to initiate therapy, based on delivered fractions. Reasons for non-completion (financial, logistic, social) were recorded qualitatively.

Patients were followed in outpatient clinics and by telephone. The primary outcome was overall survival, defined as time from surgery to death or last contact (censored). Patients who were alive at the time of last follow-up were considered right-censored at that date. Survival was estimated using Kaplan-Meier curves and compared with the log-rank test. Cox proportional hazards models explored prognostic variables including age, tumor size, extent of resection, and adjuvant therapy completion. Given the very small STR subgroup and early clustering of events among patients who did not initiate adjuvant therapy, a parsimonious multivariable model was applied to avoid over-fitting. All statistical analyses were performed using SPSS Statistics for Windows, Version 24.0 (IBM Corp., Armonk, NY, USA). Statistical significance was set at a p-value <0.05.

## Results

Clinical and radiological features

The mean age was 9.7 years, with a male-to-female ratio of 2.3:1. The age range of the cohort was 3-28 years. Most patients, 53 (94.7%), were from rural backgrounds. Headache, vomiting, and ataxia were each seen in 55 (98.2%) patients, followed by papilledema in 54 (96.4%) patients (Table 1). Based on available clinical criteria, 48 (85.7%) patients were categorized as standard-risk and eight (14.3%) as high-risk.

**Table 1:** Baseline clinical, radiological, surgical, and histopathological characteristics of patients with medulloblastoma (n = 56). Data are presented as number (percentage). Percentages are calculated using the total cohort (n = 56) as the denominator.

Variable	Category	N = 56 (%)
Age in years	<10	29 (51.8%)
	10–20	18 (32.1%)
	>20	9 (16.1%)
Sex	Male	40 (71.4%)
Residence	Rural	53 (94.7%)
Clinical presentation	Headache	55 (98.2%)
	Vomiting	55 (98.2%)
	Ataxia	55 (98.2%)
	Diminution of vision	15 (26.8%)
	Papilledema	54 (96.4%)
Tumor location	Vermis	50 (89.3%)
	Cerebellar hemisphere	4 (7.1%)
	Cerebellopontine angle	2 (3.6%)
Enhancement	Heterogeneous	18 (32.1%)
Tumor size	≥50 mm	38 (67.9%)
Extent of resection	Gross total excision	47 (83.9%)
	Near-total excision	7 (12.5%)
	Subtotal resection	2 (3.6%)
Postoperative morbidity	Meningitis	3 (5.4%)
	Hemorrhage	4 (7.1%)
	Lower cranial nerve palsy	34 (60.7%)
	Mutism	4 (7.1%)
Histopathology	Classic medulloblastoma	49 (87.5%)
	Desmoplastic/Nodular medulloblastoma	6 (10.7%)
	Medulloblastoma with extensive nodularity	1 (1.8%)

MRI showed vermian origin in 50 (89.3%) patients, cerebellar hemispheric origin in four (7.1%) patients, and cerebellopontine-angle origin in two (3.6%) patients. Tumor size ≥50 mm in 38 (67.9%) patients and heterogeneous enhancement in 18 (32.1%) patients were associated with poorer survival on univariate analysis (Table 1).

Extent of resection and complications

GTR was achieved in 47 (83.9%) patients, NTR in seven (12.5%) patients, and STR in two (3.6%) patients. Postoperative complications included lower cranial nerve palsy in 34 (60.7%) patients, cerebellar mutism in four (7.1%) patients, meningitis in three (5.4%) patients, and hemorrhage in four (7.1%) patients (Table 1).

Adjuvant therapy

Combined adjuvant therapy consisting of craniospinal irradiation followed by chemotherapy was planned for all eligible patients. In total, 41 (73.2%) patients completed craniospinal irradiation (≥30 fractions) followed by chemotherapy (Group A), two (3.6%) patients discontinued early, and 13 (23.2%) patients did not initiate adjuvant therapy because of socioeconomic or logistic constraints (Table 2). All patients who did not initiate or complete adjuvant therapy died within six months.

**Table 2:** Adjuvant therapy completion and survival outcomes in the study cohort (n = 56). Median survival was 23 months in patients completing adjuvant therapy versus 4 months in those not completing or not initiating therapy (log-rank p < 0.0001). Data are presented as number (percentage). Adjuvant therapy included craniospinal irradiation with posterior fossa boost followed by chemotherapy.

Variables	N = 56 (%)
Adjuvant therapy completed	41 (73.2%)
Adjuvant therapy not completed/not initiated	15 (26.8%)
Alive at the last follow-up	18 (32.2%)
Expired	38 (67.8%)

Histopathology

The predominant histological variant was classic medulloblastoma in 49 (87.5%) patients, followed by desmoplastic/nodular in six (10.7%) patients and medulloblastoma with extensive nodularity in one (1.8%) patient (Table 1). Molecular subtyping was unavailable due to limited resources.

Postoperative outcome

Despite aggressive surgery, 38 (67.8%) patients died during follow-up. The median overall survival for the cohort was 15 months (interquartile range = 6-33 months). In total, 13 (23.2%) patients died before initiating adjuvant therapy, and two (3.6%) patients died before completing treatment, underscoring the impact of treatment attrition on survival in resource-limited settings. Only 18 (32.2%) patients were alive at the last follow-up (Table 2).

Survival outcomes

Univariate analysis also showed that the presence of ataxia was associated with longer survival (p = 0.011), tumor size ≥50 mm was associated with poorer survival (p < 0.00001), and heterogeneous enhancement was adverse (p = 0.00013). Age, sex, and hydrocephalus were not significant prognostic factors. Univariate comparisons and Cox regression results are summarized below.

**Figure 1:**
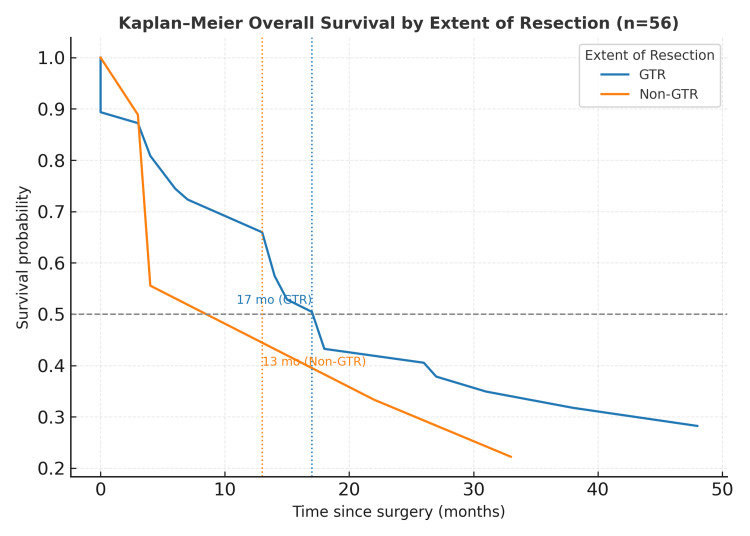
Kaplan–Meier (univariate analysis) survival curves stratified by extent of resection. Kaplan–Meier overall survival curves comparing GTR and incomplete resection (non-GTR; NTR + STR). Median survival was 17 months for GTR and 13 months for non-GTR. Log-rank (Mantel–Cox) test: χ² = 0.37, p = 0.83 → not statistically significant. GTR = gross total resection; NTR = near-total resection; STR = subtotal resection

When stratified by extent of resection (Figure 1), Kaplan-Meier estimated median survival was 17 months for patients with GTR, whereas those with incomplete resection (NTR + STR) had an estimated median survival of 13 months. The difference was not statistically significant (log-rank χ² = 0.37, p = 0.83).

**Figure 2:**
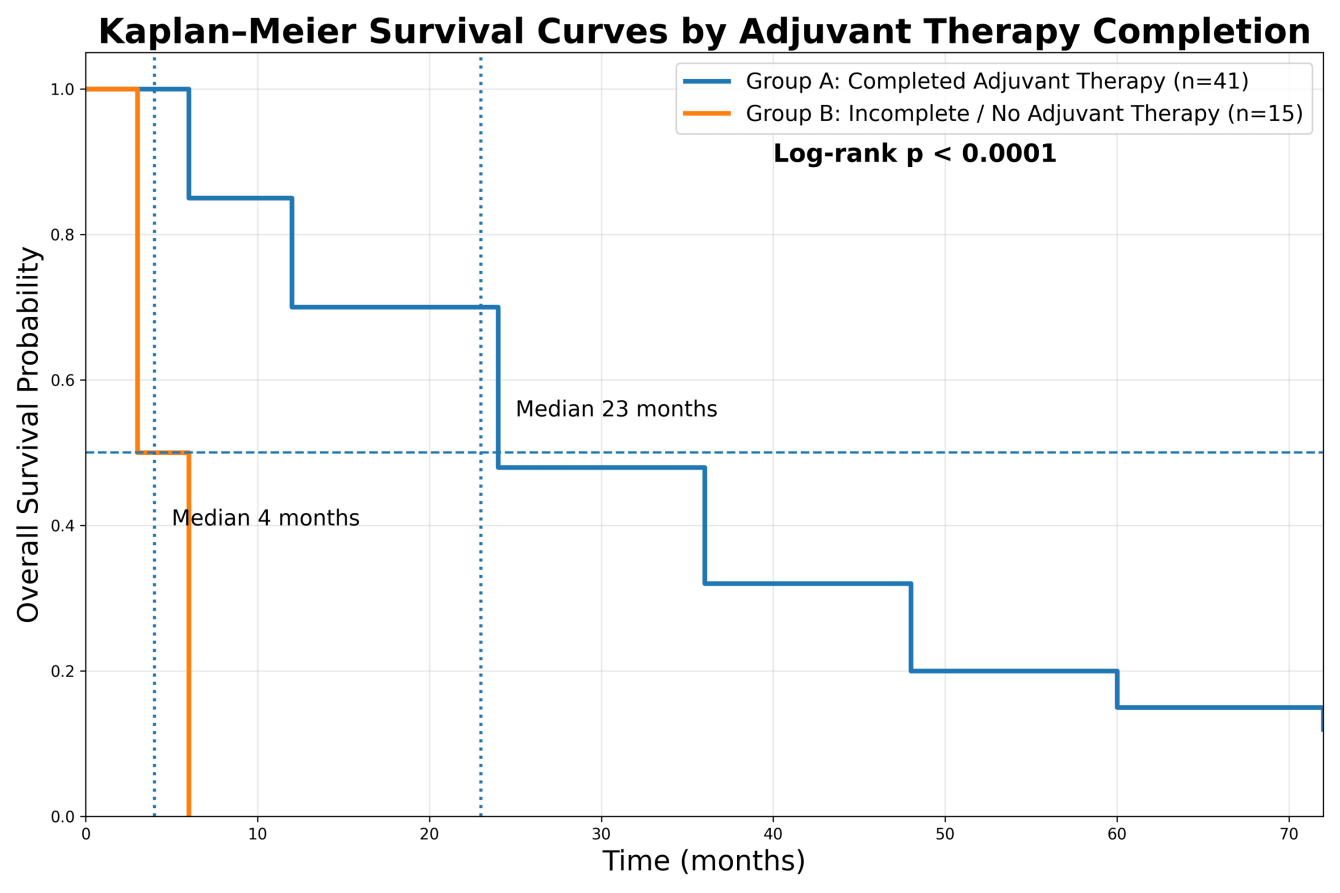
Kaplan–Meier overall survival curves stratified by completion of adjuvant therapy (craniospinal radiotherapy followed by chemotherapy). Kaplan–Meier overall survival curves comparing patients who completed adjuvant therapy (Group A) and those who did not complete or initiate therapy (Group B). Median survival was 23 months versus 4 months, respectively. The difference was statistically significant (log-rank p < 0.0001).

Completion of adjuvant therapy showed marked survival separation on Kaplan-Meier analysis (Figure 2). Kaplan-Meier estimated median survival was 23 months for patients completing adjuvant therapy (Group A) compared to four months in Group B (p < 0.0001). However, Cox regression did not demonstrate independent significance (hazard ratio = 0.98, 95% confidence interval = 0.65-1.47, p = 0.95), likely reflecting violation of the proportional hazards assumption and early clustering of deaths among patients who did not initiate adjuvant therapy in this small cohort.

On multivariate Cox regression including age, tumor size, extent of resection, and adjuvant therapy completion, no variable retained independent prognostic significance. The lack of significance likely reflects limited sample size, particularly the small STR subgroup and early event clustering among patients who did not initiate adjuvant therapy. Disease-free survival could not be reliably calculated due to incomplete follow-up imaging; overall survival was used as the primary endpoint.

## Discussion

Patients completing adjuvant therapy achieved a median survival of 23 months, whereas those who discontinued or did not initiate therapy survived a median of four months. Similarly, GTR yielded a median survival of 17 months versus 13 months for incomplete resections. These findings reflect the disparity between LMIC and HIC outcomes, where five-year survival typically exceeds 70% [5,6,13]. Economic constraints, delayed presentation, and poor compliance remain predominant determinants of mortality [7,9,14]. In addition to overall outcomes, the relative contribution of surgical and adjuvant treatment factors to survival was further analyzed. In terms of surgical factors, although GTR was achieved in 47 (83.9%) cases, the extent of resection did not significantly correlate with survival (p = 0.83). Keeling et al. [15] and Dhall [16] similarly noted that incomplete resection alone does not portend a worse prognosis when adjuvant therapy is optimized. In this series, the lack of impact likely reflects adjuvant non-compliance overriding surgical benefit. Nonetheless, achieving maximal safe resection remains a cornerstone of management to reduce tumor burden and improve CSF dynamics [17].

The impact of adjuvant therapy was particularly pronounced, as Kaplan-Meier analysis demonstrated a pronounced survival difference between patients completing adjuvant therapy and those who did not (p < 0.0001). Comparable LMIC studies have repeatedly emphasized this as a clinically relevant modifiable factor [8,12,18]. In the present study, the absence of significance in multivariate regression (p = 0.95) likely results from early clustering of deaths among patients who did not initiate adjuvant therapy and limited sample size. Despite this, the directional trend reinforces that completion of adjuvant therapy remains an important modifiable factor influencing outcome in this setting. Our results reaffirm that in resource-constrained settings, treatment completion, rather than surgical extent alone, has an important influence on survival. In accordance with contemporary treatment protocols, patients completing craniospinal irradiation subsequently received adjuvant chemotherapy, which is recognized as an integral component of medulloblastoma management and contributes substantially to long-term survival.

In addition to treatment-related factors, radiological characteristics also demonstrated prognostic relevance. Specifically, tumor size ≥50 mm and heterogeneous enhancement were associated with poorer survival on univariate analysis (p < 0.00001 and p = 0.00013, respectively). Larger, heterogeneously enhancing lesions often represent higher-grade biology with necrosis or microvascular proliferation [10,11,19]. Clinical presentation at diagnosis also influenced outcomes. In this context, ataxia likely reflects earlier clinical presentation rather than biological advantage (p = 0.011). Northcott et al. [20] noted that early neurological manifestations prompt surgical intervention before irreversible sequelae, improving outcomes. Histopathological patterns were also consistent with previously reported regional trends. In our cohort, classic medulloblastoma (49, 87.5%) was the predominant variant, followed by desmoplastic/nodular (6, 10.7%) and extensive nodularity (1, 1.8%), consistent with previous regional data [12]. Absence of large-cell/anaplastic histology may reflect referral bias or demographic variation. Molecular subgrouping, which refines prognostic stratification, was unavailable in this LMIC series, a common limitation where cost and access preclude routine profiling [20,21].

On further analysis, multivariate Cox regression did not identify independent prognostic factors, in line with smaller series where limited sample size and early attrition diminish statistical power [22]. Nonetheless, adjuvant therapy completion, smaller tumor size, and early presentation remain clinically meaningful correlates of survival.

These findings must be interpreted within the broader context of LMIC neuro-oncology, where challenges include delayed presentation, limited radiotherapy facilities, and high treatment abandonment [7,8,18,23]. Family-driven discontinuation due to financial strain is frequent [24]. Decentralization of oncology services, subsidized radiotherapy access, and structured follow-up programs could mitigate attrition and improve compliance. A uniform radiotherapy dosing strategy was employed in this cohort, which may limit direct comparison with risk-adapted protocols but reflects real-world practice in resource-constrained settings.

Limitations

This retrospective single-center analysis is limited by its modest sample size and lack of molecular subgrouping. Detailed chemotherapy regimen stratification and cycle-wise data were not consistently available, as some patients received part of their adjuvant treatment or follow-up care outside the primary center, resulting in incomplete documentation of subsequent cycles. Short median follow-up restricted long-term survival assessment, and early deaths among patients who did not initiate adjuvant therapy reduced multivariate power. Nevertheless, standardized surgical and radiotherapy protocols and systematic follow-up strengthen the study’s internal validity. Future multi-institutional collaborations incorporating molecular subgrouping may yield more robust prognostic insights in LMIC settings.

## Conclusions

In this LMIC cohort, completion of adjuvant therapy emerged as an important modifiable factor influencing survival. Larger tumor size and heterogeneous enhancement were associated with poorer outcomes on univariate analysis, while ataxia at presentation likely reflected earlier detection rather than biological advantage. Addressing socioeconomic and infrastructural barriers to ensure radiotherapy completion and incorporating molecular diagnostics are key to narrowing the survival gap between LMIC and HIC settings. Ensuring access to timely radiotherapy and adopting multidisciplinary LMIC-adapted protocols remain essential to bridging the survival gap with HICs.
